# The impact of early childhood grandparenting on deviant behaviors among rural junior high school students

**DOI:** 10.3389/fpsyg.2026.1819600

**Published:** 2026-06-22

**Authors:** Yipu Hao

**Affiliations:** Central China Normal University, Wuhan, China

**Keywords:** China Education Panel Survey, deviant behaviors, early childhood grandparenting, Problem Behavior Theory, rural junior high students

## Abstract

**Introduction:**

Grandparenting is a prevalent form of early childcare in rural China, yet its long-term effects on adolescent development remain controversial. This study investigates whether early childhood grandparenting, compared with parental care, influences deviant behaviors among rural junior high school students and explores the mediating mechanisms and heterogeneous effects.

**Methods:**

Using data from the China Education Panel Survey, this study analyzed a sample of 4,746 rural junior high students. Multiple linear regression and propensity score matching were employed to estimate the effect of early childhood grandparenting on deviant behaviors. Mediation analysis was conducted using the KHB method to examine four pathways derived from Problem Behavior Theory: parent-child relationship, parent-child communication, future-oriented confidence, and self-control. Heterogeneity in the effects by gender, only-child status, early family economic conditions, and preschool education was also assessed.

**Results:**

Early childhood grandparenting significantly increased deviant behaviors among rural junior high students. This effect was robust to various specification checks, including doubly robust models and omitted variable tests. Heterogeneity analyses showed stronger effects among female students, those from poorer early economic backgrounds, and those without preschool education. Mediation analysis revealed that the four mechanisms jointly explained 80.44% of the total effect, with future-oriented confidence exhibiting the largest mediating proportion (25.67%), followed by parent-child communication (23.01%).

**Discussion:**

Early childhood grandparenting poses a long-term risk of exhibiting deviant behaviors among rural adolescents, primarily by weakening the perceived environment and personality systems within Problem Behavior Theory. These findings highlight the need for targeted interventions that enhance parent-child communication and strengthen future-oriented confidence, particularly for economically disadvantaged children and those without preschool education.

## 1 Introduction

Intergenerational childcare has become a widespread phenomenon in rural China. The proportion of primary caregivers who are grandparents is 26.7% in rural areas, compared with 9.7% in urban areas (Department of Family Affairs, National Health and Family Planning Commission, 2016). The 2019 China Child Welfare and Protection Policy Report released by the China Institute of Philanthropy at Beijing Normal University indicates that grandparents raise 96% of rural left-behind children. Correspondingly, in grandparent-reared households, the prolonged absence of parental roles often results in insufficient care and nurturing in daily life, as well as a lack of supervision and guidance in behavioral development (Zhang et al., 2025). This can easily lead to developmental delays in rural children across multiple domains, including cognitive and social behaviors (Song et al., 2025). Issues related to grandparent-reared children have garnered extensive attention from researchers.

The junior high school stage is a critical period for cognitive development and psychological growth, as well as a pivotal phase for the development of social behaviors (Rose et al., 2026). The social behaviors development of junior high students not only shapes their future life paths but also impacts the sustainable development of the economy and society (Konrath et al., 2025). Deviant behaviors among junior high students creates cumulative disadvantages for their subsequent development. Such behaviors significantly increases the likelihood of alcohol abuse, violence, and criminal activity in adulthood (van Lier et al., 2012). Reducing deviant behaviors among junior high students can enhance their academic performance, influence individual human capital accumulation, and ultimately have positive effects on future educational attainment and career achievements (Segal, 2013).

Given this, this paper attempts to analyze the impact of early childhood grandparenting on deviant behaviors among rural junior high school students compared to parental care. Based on data from the China Education Panel Survey, this study examines the effects of early childhood grandparenting on junior high students’ deviant behaviors, focusing on three key questions: First, does early childhood grandparenting influence deviant behaviors among rural Chinese junior high students and undermine rural human capital accumulation? Second, what mechanisms mediate the effect of early childhood grandparenting on deviant behaviors among rural Chinese junior high students? Third, do group differences (e.g., gender) exist in the impact of early childhood grandparenting on deviant behaviors among rural Chinese junior high students?

This study makes several distinctive contributions to the existing literature. First, while most prior research focuses on the immediate effects of grandparenting, this study investigates its long-term impact on deviant behaviors during early adolescence, thereby extending the temporal horizon of evidence. Second, unlike previous studies that often examine isolated mediating factors, this study integrates the perceived environment system (parent–child relationships and communication) and the personality system (future-oriented confidence and self-control) from Problem Behavior Theory (PBT) into a unified analytical framework, allowing for a comparative assessment of their relative explanatory power. Third, this study systematically explores heterogeneous effects across gender, only-child status, early family economic conditions, and preschool education, offering nuanced insights for targeted interventions. These innovations not only advance theoretical understanding of how early grandparenting shapes adolescent outcomes but also provide empirical foundations for policy design in rural China.

## 2 Literature review

### 2.1 The impact of early childhood grandparenting on deviant behaviors

Developed by American psychologist Jessor (2014), Problem Behavior Theory provides a classic framework for understanding adolescent problem behaviors, including alcohol and drug use, and delinquency. According to PBT, these behaviors emerge from the interaction of three systems: the perceived environment system (e.g., family structure, school climate, peer influence), the personality system (self-identity, risk perception, coping styles), and the behavioral system, which generates a dynamic cycle of choices and social consequences (Jessor, 2014). This multi-system perspective offers an integrative lens to examine how early childhood grandparenting shapes students’ deviant behaviors (Jessor and Jessor, 1977).

PBT has been widely employed globally. In Western contexts, it has clarified determinants of smoking, drinking, substance use, and delinquency, informing prevention programs (Vazsonyi, 2008). Chinese researchers have adapted it to investigate future smoking intentions among youths (Cai et al., 2015).

According to PBT, students’ deviant behaviors result from multiple overlapping social factors, with family being a critical risk factor (Richard Jessor, 1991). At the same time, life course theory emphasises that students’ developmental levels depend not only on the socioeconomic environment during childhood but also on the social resources and opportunities available within the constraints of historical contexts and social systems throughout their growth (Elder, 1998). From a life cycle perspective, the early life course is a critical period for human capital formation, and the quality of human capital formed during this stage influences the returns on human capital investment throughout the entire life cycle (Heckman, 2000). Early childhood grandparenting experiences are embedded at the outset of an individual’s life course, becoming a significant component of their life history and exerting long-term effects (Mosley-Johnson et al., 2019). Therefore, early childhood grandparenting may be a significant factor influencing students’ deviant behaviors.

At present, academic circles have yet to reach a consensus on whether intergenerational childcare primarily exerts a positive or negative influence on student behaviors compared to parental care. This divergence may stem from variations in survey data, inconsistent indicator selection and definition, or differing research methodologies. According to the developmental resource model (Chang et al., 2019), the family is the most crucial external resource for students’ behavioral development. Grandparent-led childcare leads to the depletion of developmental resources, such as the child’s social relationships with parents, thereby weakening the essential conditions for student development and increasing the likelihood of deviant behaviors (Theokas and Lerner, 2006). Comparative studies of children aged 3–7 reveal higher rates of deviant behaviors among those raised by grandparents than those raised by biological parents (Wang Y. et al., 2024). Research indicates that, compared to parental care, grandparent-led upbringing significantly negatively predicts overall self-directed behaviors and academic self-direction in children aged 6–12 (Ling et al., 2021). Teachers also commonly report that grandparent-reared children exhibit more emotional and behavioral problems compared to those raised by their parents (Edwards and Daire, 2006).

However, other studies suggest that, compared to care provided by parents, grandparent rearing has no negative impact on child development and may even yield positive effects. Studies in India and South Africa found that, compared to parental care, children raised by grandparents had lower rates of depression and higher levels of prosocial behaviors (Profe and Wild, 2017; Renny and Jayasankara, 2016). A survey in China also found that, compared to parental care, children raised by grandparents tended to develop characteristics such as relative independence, thriftiness, and politeness (Tadesse et al., 2025). A review of existing research indicates that most studies support the notion that grandparenting increases children’s deviant behaviors compared to parental care. However, this conclusion primarily stems from studies examining the immediate effects of grandparenting on student behaviors. Waldfogel (2007) notes that the impact of non-parental caregiving varies with children’s age. Therefore, it is necessary to examine the effects of grandparenting within the developmental trajectory of childhood and to explore the long-term impact of early childhood grandparenting on student behaviors compared to parental care. Based on this, the following hypothesis is proposed: H1: early childhood grandparenting is positively correlated with deviant behaviors among rural junior high school students.

### 2.2 Mechanisms through which early childhood grandparenting influences deviant behaviors

Although an increasing body of literature has documented the immediate effects of grandparenting on behaviors, few studies have systematically examined, within a unified theoretical framework, the long-term mediating pathways through which early childhood grandparenting shapes adolescent deviant behaviors. Existing research often focuses in isolation on family relationship factors or individual personality traits, resulting in an insufficient exploration of the relative importance of these two mechanisms (Wang Y. et al., 2024; Zeng et al., 2024). By integrating the “perceived environmental system” and the “personality system” from “PBT” into a single analytical model, this study fills this research gap, thereby elucidating how early childhood grandparenting indirectly increases deviant behaviors through multiple distinct pathways.

Within the PBT framework, the perceived environment system refers to adolescents’ subjective perceptions and evaluations of their social environments, including family, school, and peer contexts. Jessor (1991) distinguishes between distal structures (e.g., social support, control levels) and proximal structures (e.g., satisfaction with family relationships, peer pressure, parental involvement) (Bonino et al., 2005). Proximal structures are directly relevant to daily interactions and have a more immediate influence on behavioral choices PBT holds that how students perceive environment system is a crucial factor in shaping their conduct. Bonino et al. (2005) highlight that the perceived environmental system serves as a mediator linking family to adolescent behaviors.

#### 2.2.1 Mechanism 1: Perceived environment system (parent–child relationships and parent–child communication)

According to PBT, early childhood grandparenting weaken parent–child relationships and reduce parent–child communication, which in turn can lead to deviant behaviors. For children raised by grandparents during early childhood, the physical and emotional distance from parents can have lasting consequences for the parent–child relationship. Family systems theory similarly posits that interactions among components within the family system mean that a single characteristic—such as the absence of parents during early years—can influence the entire family system or its subsystems (Weeland et al., 2021). During the critical early years, children who do not establish deep parent–child bonds with their mothers and fathers may find it difficult to form such bonds later, even after returning to parental care (Fergusson et al., 2008). As a result, parent–child relationship quality in adolescence tends to be significantly lower among those who experienced early grandparenting.

Coleman (1988) conceptualized the bond between parents and children as a form of “social capital” that is crucial for students’ socialization and development. Empirical research consistently shows that poor parent–child relationships are significant predictors of deviant behaviors, delinquency, and aggression (Sentse and Laird, 2010; Kheradmand et al., 2018). Conversely, warm and supportive parent–child relationships serve as protective factors that reduce the likelihood of adolescent problem behaviors.

In addition to the quality of the relationship, the frequency and depth of parent–child communication are also affected by early grandparenting. In grandparent-rearing households, parents often live apart from their children for extended periods. Even when they remain in contact, communication may focus narrowly on academic performance and material needs, while neglecting the child’s psychological and emotional concerns (Pan and Ye, 2017). This pattern can persist into adolescence, leading to chronically reduced communication. Prior research has demonstrated that parent–child communication exhibits a significant negative correlation with students’ behavioral and emotional problems (Kapetanovic et al., 2019). Good communication facilitates the transmission of parental values, enhances monitoring, and provides a channel for emotional support, all of which reduce deviant behaviors. Therefore, we propose: *H2a: Early childhood grandparenting influences deviant behaviors through the perceived environment system, specifically by weakening parent–child relationships and reducing parent–child communication*.

#### 2.2.2 Mechanism 2: The personality system (future-oriented confidence and self-control)

The personality system in PBT consists of three main components: the motivational structure (e.g., future-oriented confidence, academic aspirations), the personal belief structure (e.g., values, attitudes toward deviance), and the personal control structure (e.g., self-control, risk-taking propensity, emotional stability) (Karaman, 2013). These personality factors shape how adolescents interpret environmental stimuli and whether they resort to deviant behaviors as coping mechanisms.

Future-oriented confidence is a key element of the motivational structure. It refers to an adolescent’s internal belief that they can achieve their long-term goals and that their efforts will lead to positive outcomes (Wang et al., 2026). Adolescents with strong future confidence are more likely to engage in goal-directed behaviors (e.g., studying, career planning) and are less likely to seek short-term gratification through risky or deviant acts (Richard Jessor, 1991). Conversely, when future confidence is low, adolescents may feel that there is little point in investing in conventional pursuits, making them more susceptible to the appeal of immediate rewards offered by deviant behaviors (Gouveia-Pereira et al., 2017).

Early childhood grandparenting can undermine future-oriented confidence through several pathways. First, grandparents often have lower educational levels and may hold less ambitious expectations for their grandchildren compared to parents (Mortimer and Lee, 2022). Second, the absence of parental role models who demonstrate goal-directed behaviors may leave children with fewer examples of how to pursue long-term success (Hill and Tyson, 2009). Third, the reduced emotional support and supervision from parents can lead to a general sense of insecurity and pessimism about the future (Eisman et al., 2015). Thus, we expect that early grandparenting will be associated with lower levels of future-oriented confidence, which in turn will increase deviant behaviors.

Self-control is the centerpiece of the personal control structure within PBT. Self-control refers to the ability to regulate one’s emotions, impulses, and behaviors in accordance with internal standards and social norms (Wang et al., 2026). Adolescents with poor self-control are more likely to exhibit emotional volatility, such as anxiety, irritability, or impulsivity (Vazsonyi et al., 2017). They have difficulty delaying gratification and are more prone to risk-taking behaviors, including fighting, substance use, and rule-breaking (Vazsonyi and Huang, 2010). Research from China has shown that self-control mediates the relationship between parenting styles and adolescent externalizing problem behavior (Zhang and Wang, 2023).

Early childhood grandparenting can impair the development of self-control (Zheng et al., 2025). Parents typically engage in more consistent and structured discipline, set clear behavioral expectations, and provide immediate feedback for rule violations. Grandparents, especially those who are older or less educated, may adopt more permissive or inconsistent parenting styles (Lytton, 1977; Yang et al., 2024). They may spoil grandchildren out of affection or avoid confrontation, thereby failing to teach essential self-regulatory skills. Moreover, the reduced parental monitoring associated with grandparenting means that deviant impulses are less likely to be corrected in real time, allowing poor self-control habits to consolidate (Liu and Vazsonyi, 2024). Consequently, we hypothesize that early grandparenting leads to lower self-control, which subsequently increases deviant behaviors. Based on this reasoning, we propose: *H2b: Early childhood grandparenting influences deviant behaviors through the personality system, specifically by lowering future-oriented confidence and diminishing self-control*.

Despite the valuable insights provided by existing studies, several critical gaps remain. First, most prior research focuses on the immediate effects of grandparenting on child behaviors, leaving the long-term impact of early childhood grandparenting on adolescent outcomes largely underexplored. Second, existing studies often examine mediating mechanisms in isolation, without comparing their relative importance within a unified theoretical framework. This fragmentation limits our understanding of which pathway is most influential. Third, the literature has paid insufficient attention to heterogeneous effects across key subgroups such as gender, early economic conditions, and preschool education, which are essential for designing targeted interventions. Fourth, methodological limitations—particularly self-selection bias due to observable differences between early childhood grandparenting and parental care—are often inadequately addressed. To address these shortcomings, this study conducts an exploratory analysis.

## 3 Methods

### 3.1 Data

The data primarily originate from the 2014–2015 academic year dataset of the China Education Panel Survey (CEPS). CEPS, designed and implemented by the China Survey and Data Center at Renmin University of China, is currently the largest publicly available, nationally representative specialized education survey. Its objectives are to reveal the influence of family, school, community, and macro-social structures on individual educational outcomes and to explore further the processes through which these outcomes exert their effects across the individual life course. The 2014–2015 survey serves as a follow-up to the 2013–2014 baseline survey, tracking students who were in the first year of junior high school (Grade 7) at the time. This represents the most recent publicly available data from CEPS.

During data processing, this paper first horizontally merged student and parent data by sample code, then longitudinally merged them with baseline survey data. For research purposes, this paper retained only successfully followed-up eighth-grade student samples, excluding unsuccessful follow-ups and new samples, and ultimately retained only those with rural household registrations. After applying listwise deletion to remove samples with missing values, the final valid sample size was 4,746.

### 3.2 Measures

#### 3.2.1 Dependent variable: deviant behaviors

Deviant behaviors among rural junior high students serves as the dependent variable. There behaviors are measured using 10 questions from the CEPS questionnaire, each beginning with “In the past year, have you engaged in the following behaviors?” These questions encompass 10 sub-items covering: “cursing or using foul language,” “arguing,” “fighting,” “bullying weaker classmates,” “having a short temper,” “inability to concentrate,” skipping class, truancy, or dropping out,” “copying homework or cheating on exams,” “smoking or drinking alcohol,” and “visiting internet cafes or arcades.” This study assigned values of 1–5 to the response options “never, rarely, sometimes, often, always.” The scores from all 10 questions were then summed to create a deviant behaviors variable for rural junior high students, ranging from 10 to 50. Higher scores indicate more frequent occurrence of deviant behaviors among rural junior high students. The scale has a Cronbach’s alpha coefficient of 0.821.

#### 3.2.2 Independent variable: early childhood grandparenting

The core explanatory variable is early childhood grandparenting, defined by responses to the CEPS question: “Before the child entered elementary school, who was primarily responsible for their care?” Respondents who answered “child’s grandparents” or “child’s maternal grandparents” were assigned a value of 1 for the “early childhood grandparenting” variable; those who answered “child’s parents” were assigned a value of 0. Due to the small sample size for the other options (“non-parent and (maternal/paternal) grandparent relatives,” “non-relatives,” and “nannies”), these cases were excluded for research convenience.

#### 3.2.3 Mediating variables

Parent-child relationships encompass both father-child and mother–child bonds. This study measured these relationships based on the student questionnaire items “How is your relationship with your father?” and “How is your relationship with your mother?” The corresponding response options “Not close,” “Average,” and “Very close” were assigned values of 1, 2, and 3, respectively. The scores from both questions were then summed; higher scores indicate stronger parental bonds. The scale has a Cronbach’s alpha coefficient of 0.625.

Parent-child communication similarly encompasses interactions with both father and mother. The questionnaire specifically asked, “Do your parents often discuss the following topics with you?” covering five areas: school events, relationships with friends, relationships with teachers, personal feelings, and personal concerns or worries. This study also employs positive scoring, assigning values of 1–3 to the options “Never,” “Occasionally,” and “Frequently,” respectively. The scores from each parent’s five questions are then summed. Higher values indicate more frequent parent–child communication. The scale has a Cronbach’s alpha coefficient of 0.868.

Future-oriented confidence: This variable was measured using a single item from the student questionnaire: “Do you have confidence in your future?” Responses of “not confident at all=1, not very confident=2, relatively confident=3, very confident=4”.

Self-control. This is assessed using three questions, such as “Even though there are other reasons, I will still try my best to go to school, do my best to complete assignments that take a long time, and do my best to complete assignments I don’t like.” A 4-point Likert scale is used, with responses ranging from “Strongly Agree” (1) to “Strongly Disagree” (4), reflecting the level of self-control. The scale has a Cronbach’s alpha coefficient of 0.651.

#### 3.2.4 Control variables

To more scientifically assess the impact of early childhood grandparenting on deviant behaviors among rural junior high students, this study further incorporated student-level and family variables as controls. Specifically, student characteristics included age, gender, only-child status, current family socioeconomic status, early family economic conditions, preschool attendance, parental educational expectations, two-parent families, parental relationship (good = 1; others = 0), boarding status, and self-rated health. Additionally, to control for school and class factors, this study included class fixed effects.

### 3.3 Statistical analysis

#### 3.3.1 OLS regression

To analyze the impact of early childhood grandparenting on deviant behaviors among rural junior high school students, this study constructs the following benchmark regression equation estimated using ordinary least squares (OLS):


$$\text{deviant}\_\mathrm{be}h_{\mathrm{st}}=\beta_{j0}+\beta_{1}\text{early}\_\text{grandparenting}+\sum_{i=2}^{i=n}\beta_{i}X_{\mathrm{st}}+\emptyset_{s}+\varepsilon_{\mathrm{st}}$$

Where *subscripts s and* t denote the *s*-th class and the *t*-th student, respectively, 𝑋 represents individual and household control variables, $\emptyset_{s}$ denotes class fixed effects to control for school and class factors such as classroom atmosphere, and ε is the error term.

#### 3.3.2 Propensity score matching method

A major concern in estimating the causal effect of early childhood grandparenting is self-selection bias. Families who choose grandparenting may differ systematically from those who choose parental care on observable characteristics (e.g., socioeconomic status, parental education, geographic location). If these observable characteristics also affect deviant behavior, OLS estimates may be biased. To address this concern, we employed Propensity Score Matching (PSM), as developed by Rosenbaum and Rubin (1983).

The PSM approach involves three steps. First, we estimated a logit model to compute the propensity score-the predicted probability of receiving treatment (i.e., early childhood grandparenting) conditional on observable pre-treatment covariates X . Second, we matched each treated student (early grandparenting) with one or more untreated students (parental care) who had similar propensity scores. We employed four matching algorithms to ensure robustness: nearest-neighbor matching, radius matching, kernel matching, and Mahalanobis distance matching. Third, after confirming that the matching successfully balanced the covariate distributions between treatment and control groups, we computed the Average Treatment Effect on the Treated (ATT) as: $\mathrm{ATT}=E(Y_{i}^{T}|D_{i}=1,\mathrm{Pr}(X))-E(Y_{i}^{C}|D_{i}=0,\mathrm{Pr}(X))$ . where $Y_{i}^{T}$ is the deviant behaviors of student i under treatment, $Y_{i}^{C}$ under control, $D_{i}=1$ for early grandparenting, and Pr(X) is the propensity score. The ATT represents the effect of early childhood grandparenting on those who actually experienced it.

### 3.4 Descriptive statistics

Descriptive statistics are presented in Table 1. Among the analyzed sample, adolescents raised by grandparents in early childhood accounted for 23.7% of the total sample. A simple comparison of columns 2 and 4 in Table 1 reveals that adolescents raised by grandparents in early childhood scored higher on deviant behaviors than those raised by parents, and also reported relatively poorer self-rated health. Regarding individual and family characteristics, a slightly higher proportion of males than females were raised by parents, and the proportion of only-children raised by grandparents was higher than that of non-only children. Adolescents raised by grandparents experienced poorer early family economic conditions compared to those raised by parents, had slightly lower rates of preschool education, and reported poorer parental relationships. Comparing the characteristics of grandparent-reared and parent-reared adolescents reveals differences across multiple dimensions, suggesting a self-selection issue in early childhood grandparenting that warrants attention and resolution in future research.

**Table 1:** Descriptive Statistics

Variables name	Early childhood grandparenting		Parental Care	
	Mean	Standard Deviation	Mean	Standard Deviation
Deviant behaviors	14.813	4.284	14.392	4.139
male	0.510	0.500	0.529	0.499
Age	13.313	0.464	13.377	0.485
Only-child	0.295	0.456	0.266	0.442
Self-Rated Health	3.774	0.918	3.844	0.936
Boarding	0.482	0.500	0.451	0.498
Received preschool education	0.774	0.418	0.781	0.413
Parental Educational Expectations	16.197	3.284	16.304	3.341
Family socioeconomic status	39.773	11.371	38.887	11.514
Early Family Economic Conditions	2.585	0.681	2.620	0.693
Parental Relationship	0.850	0.357	0.868	0.339
two-parent families	0.639	0.481	0.844	0.362
Parent-Child Communication	7.958	2.120	8.255	2.050
Parent-Child Relationship	5.018	0.997	5.270	0.882
Future-oriented confidence	2.695	0.499	3.102	0.670
Self-control	12.317	2.509	12.739	2.680
N	1,126		3,620	

## 4 Results

### 4.1 Benchmark model: OLS estimation

Table 2 presents the estimation results for Model 1. Without controlling for other variables, early childhood grandparenting exerts a significant positive effect on adolescent deviant behaviors compared to parental care, suggesting that this grandparenting is associated with increased deviant behaviors among rural junior high school students. After further controlling for a series of variables, including individual and family characteristics, the estimated coefficient for early childhood grandparenting’s impact on rural junior high students’ deviant behaviors decreased. Still, its significance remained unchanged—Model 3 further controls for school-level fixed effects, as in Model 2. The results show that the explanatory power of the model is effectively enhanced. Although the coefficient for early childhood grandparenting becomes smaller, its influence remains statistically significant.

**Table 2:** Effects of early childhood grandparenting on deviant behaviors.

Variable	Model 1	Model 2	Model 3
Early childhood grandparenting	0.420^**^ (0.145)	0.349^**^ (0.146)	0.346^**^ (0.147)
Male		1.276^**^ (0.117)	1.176^**^ (0.117)
Age		0.003 (0.125)	−11.179^**^ (0.740)
Only-child		−0.561^**^ (0.135)	−0.272^*^ (0.148)
Self-rated health		−0.273^**^ (0.072)	−0.226^**^ (0.070)
Boarding		0.337^**^ (0.128)	0.259 (0.229)
Receiving preschool education		−0.277^*^ (0.152)	−0.191 (0.154)
Parental educational expectations		−0.155^**^ (0.022)	−0.139^**^ (0.022)
Family socioeconomic status		0.001 (0.007)	0.013^*^ (0.007)
Early family economic conditions		−0.142 (0.097)	0.108 (0.098)
Parental relationship		−1.236^**^ (0.213)	−1.181^**^ (0.214)
Two-parent families		−0.403^**^ (0.175)	−0.247 (0.192)
Class fixed effects	Uncontrolled	Uncontrolled	Controlled
*N*	4,746	4,746	4,746
Adj. *R*^2^	0.002	0.067	0.117

1. Values in parentheses indicate robust standard errors; 2. ^*^p < 0.1; ^**^p < 0.05; ^***^p < 0.01.

### 4.2 Propensity score matching method

As shown in Table 1, rural junior high students raised by parents and those raised by grandparents exhibit numerous differences in personal and family characteristics. This suggests that the decision to engage in grandparenting may not be random but rather self-selective, potentially introducing bias into OLS regression results. Therefore, propensity score matching is employed to address this issue.

The study presents estimation results from four propensity score matching (PSM) methods, including nearest neighbor matching (*k* = 4, one-to-four matching with no replacement), radius matching (*r* = 0.01), kernel matching (using the Epanechnikov kernel with a bandwidth of 0.06), and Mahalanobis distance matching. Table 3 presents the average treatment effect on the treated (ATT) obtained using these four matching methods. The results indicate that all ATT values are significantly positive. At the same time, the Average Treatment Effect (ATE) estimates obtained from all matching methods are also significantly positive. This suggests that rural junior high students who were raised by their grandparents during early childhood exhibit higher levels of deviant behavior compared to those raised by their parents. Appendix Figures A1 and A2 show the propensity score distributions for the experimental and control groups before and after matching. After matching, there is a large overlap in the common support intervals of the propensity scores, indicating that self-selection bias should not pose a serious problem. The results of the tests in Appendix Table A1 show that the standard errors for all groups after matching are below 5%, and the t-tests for all covariates between the treatment and control groups are not significant, satisfying the requirements of the balance test. Furthermore, drawing on Rubin’s proposed methodology, we comprehensively examine sample equilibrium and matching quality by assessing pseudo *R*^2^, likelihood ratio (LR), mean bias, median bias, *B*-statistic, and *R*-statistic (Rubin, 2001). As shown in the test results in Appendix Table A2, all values decreased significantly after data matching, indicating that the overall balance requirement was satisfied.

**Table 3:** Effects of early childhood grandparenting on deviant behaviors (ATT and ATE).

Matching method	ATT	ATE
Nearest neighbor matching	0.393^**^ (0.165)	0.484^***^ (0.123)
Radius matching	0.308^**^ (0.152)	0.391^***^ (0.119)
Kernel matching	0.279^*^ (0.151)	0.413^***^ (0.122)
Mahalanobis distance matching	0.450^***^ (0.156)	0.426^**^ (0.176)

1. Values in parentheses indicate robust standard errors; 2. ^*^p < 0.1; ^**^p < 0.05; ^***^p < 0.01.

### 4.3 Robustness test

#### 4.3.1 Doubly robust models

Given that PSM can only address self-selection issues and cannot effectively resolve problems such as model specification errors and inappropriate variable selection. The inverse probability weighting regression adjustment model (IPWRA) and the augmented inverse probability weighting model (AIPW) both combine regression adjustment with inverse probability weighting estimation. Their advantage lies in not requiring that both the outcome and selection models be perfectly specified simultaneously. As long as one of them is correctly specified, the estimation results remain robust, hence their designation as doubly robust models (Linden et al., 2016). To overcome the limitations of PSM methods, this study employs IPWRA and AIPW models to examine the impact of early childhood grandparenting on deviant behavior among rural junior high school students. Table 4 shows that both IPWRA and AIPW estimates of the average treatment effect indicate a significant positive association between early childhood grandparenting and deviant behavior among rural junior high school students. Furthermore, to validate the robustness of these findings, regression-adjusted model (RA) estimates are reported, which similarly confirm the reliability of the study’s conclusions.

**Table 4:** The impact of early childhood grandparenting on deviant behavior (doubly robust models).

Model	ATT	Standard error	*T*-value
IPWRA model	0.415 ^***^	0.148	2.80
AIPW model	0.416^***^	0.148	2.80
RA model	0.403^***^	0.147	2.74

^*^p < 0.1; ^**^p < 0.05; ^***^p < 0.01.

#### 4.3.2 Omission variable effect test

The preceding OLS regression, propensity score matching, and double-robust models all analyzed the impact of early grandparenting on deviant behaviors among rural junior high students using observable variables. Results consistently indicate that early grandparenting exerts a significant adverse effect on such behaviors. Although propensity score matching and the double robust model effectively address self-selection bias caused by observable variables, they cannot address endogeneity stemming from omitted variables, which may also affect the accuracy of this study’s findings. To examine the potential impact of omitted variables on the aforementioned conclusions, this study employs Oster’s method for further robustness testing (Oster, 2019).

When estimating *the* actual coefficient β^*^using Oster’s method, the results depend on two parameters: the ratio *δ* between the explanatory power of observable variables and that of unobservable omitted variables over the dependent variable, and the maximum goodness-of-fit *R*_max_ of the regression equation if all unobservable factors were included in the model. Oster further proposes a specific testing method: (1) Assuming the strength of the relationship between the observed variables, omitted variables, and the dependent variable is identical (*δ* = 1), if given the value of *R*_max_ (Oster recommends using 1.3 times the current regression’s *R*^2^, i.e., increasing the regression equation’s goodness-of-fit by 1.3 times) and a *δ* value (Oster recommends 1), the estimated independent variable β^*^ should fall within the 95% confidence interval of the *β* estimate from the basic regression results. This indicates the robustness of the regression results. (2) After increasing the goodness-of-fit of the regression equation by a factor of 1.3, If the implied δ exceeds 1 when assuming *β* = 0, this indicates that the correlation between the observed variables and the independent variable is stronger than the correlation between the omitted variable and the independent variable. This confirms the robustness of the regression results.

Table 5 presents the results of the omitted variable test. Regarding the effect of early childhood grandparenting on deviant behaviors among rural junior high students, the β* estimate from Method 1 falls within the 95% confidence interval of the basic regression estimate, indicating that the test is passed. The *δ* value calculated by Method 2 is 7.2, exceeding the standard threshold of 1. This suggests that the influence of early childhood grandparenting on deviant behaviors among rural junior high students passes both tests. Consequently, after accounting for omitted-variable bias, the study’s conclusions remain robust.

**Table 5:** Omission variable test.

Test method	Testing standard	Actual calculation result	Pass/Fail
Method 1	[0.057, 0.634]	0.317	Yes
Method 2	*δ* > 1	7.20	Yes

#### 4.3.3 Nonparametric permutation test

To verify that the estimation results are not coincidental, a placebo test was conducted by randomly assigning students to either the early childhood grandparenting group or a control group, then simulating and re-regressing the estimated impact of early childhood grandparenting on behavioral problems among rural junior high students. This procedure was repeated 500 times, yielding 500 re-estimated placebo regression coefficients. The results indicate that the estimated coefficients for early childhood grandparenting are usually distributed around zero, with zero probability of exceeding the benchmark (see Appendix Figure A3). This confirms that the study’s findings are not random chance and are statistically reliable (Li et al., 2016).

### 4.4 Mechanism analysis and heterogeneity analysis

#### 4.4.1 Mechanism analysis

Previous analysis indicates that early childhood grandparenting increases the incidence of deviant behavior among rural junior high students. However, a question warranting further consideration is: How does early childhood grandparenting influence the occurrence of deviant behaviors among rural junior high students? To address this, this study first employs the stepwise regression method proposed by Baron and Kenny to examine the influence mechanism (Baron and Kenny, 1986). The results are shown in Table 6. Early childhood grandparenting significantly reduces parent–child relationships, parent–child communication, future-oriented confidence, and self-control. When these mediators are included in the model for deviant behaviors, parent–child relationships, communication, future-oriented confidence, and self-control all exhibit significant negative effects on deviant behaviors. Importantly, the direct effect of early childhood grandparenting on deviant behaviors becomes non-significant, and its coefficient decreases compared to the baseline model. These findings indicate that early childhood grandparenting indirectly increases deviant behaviors among rural junior high students by impairing parent–child relationships, communication, future-oriented confidence, and self-control.

**Table 6:** Mechanism testing.

Variable	Parent–child relationships	Parent–child communication	Future-oriented confidence	Self-control	Deviant behaviors
Early childhood grandparenting	−0.418^**^ (0.084)	−0.243^**^ (0.039)	−0.350^**^ (0.024)	−0.280^**^ (0.110)	0.047 (0.169)
Parent–child relationships					−0.143^**^ (0.033)
Parent–child communication					−0.267^**^ (0.076)
Future-oriented confidence					−0.177^*^ (0.100)
Self-control					−0.217^**^ (0.024)
Control variables	Control	Control	Control	Control	Control
*N*	4,746	4,746	4,746	4,746	4,746
Adjusted *R*^2^	0.168	0.164	0.156	0.105	0.195

1. Values in parentheses indicate robust standard errors; 2. ^*^p < 0.1; ^**^p < 0.05; ^***^p < 0.01.

This study investigated the mediating roles of parent–child relationships, parent–child communication, future-oriented confidence, and self-control in the link between early childhood grandparenting and deviant behaviors among rural junior high students, using the KHB mediation test method (Kohler et al., 2011). Table 7 results indicate that after incorporating the four mediating variables, the direct effect of early childhood grandparenting became non-significant. The total indirect effect was significant and accounted for 80.44% of the total effect. Specifically, the mediating effects of parent–child relationships, parent–child communication, future-oriented confidence, and self-control were 18.10, 23.01, 25.67, and 13.66%, respectively. Among these, future-oriented confidence exerted the largest mediating effect, followed by parent–child communication.

**Table 7:** Mechanism analysis using KHB method.

Path	Coefficient	Standard error	Effect size (%)
Total effect	0.329^**^	0.162	
Direct effect	0.064	0.168	
Total indirect effect	0.264^***^	0.021	80.44
Parent–child relationships	0.060^***^	0.019	18.10
Parent–child communication	0.076^***^	0.023	23.01
Future-oriented confidence	0.084^***^	0.038	25.67
Self-control	0.045^***^	0.023	13.66

^*^p < 0.1; ^**^p < 0.05; ^***^p < 0.01.

#### 4.4.2 Heterogeneity analysis

The preceding section examined the overall impact of early childhood grandparenting on deviant behaviors among rural junior high students. However, whether this effect exhibits group heterogeneity requires further analysis. To address this question, this study continues to employ subgroup regression to explore potential heterogeneity in the effects of early childhood grandparenting. Table 8 presents the impact of early childhood grandparenting on deviant behaviors among rural junior high students, stratified by gender, whether an only-child or not, early family socioeconomic status, and preschool attendance.

**Table 8:** Heterogeneity analysis results.

Grouping variables	Coefficient	Standard error	*N*	Adjusted *R*^2^
Male	0.371	0.241	2,489	0.085
Female	0.384^**^	0.177	2,257	0.134
Only-child	0.445^*^	0.268	1,294	0.140
Non-only-child	0.396^*^	0.239	3,452	0.085
Received preschool education	0.312^*^	0.165	3,701	0.102
Did not receive preschool education	0.717^**^	0.363	1,045	0.159
Good early family economic conditions	1.289	1.935	569	0.060
Early family poor economic conditions	0.280^*^	0.147	4,177	0.119

Control variables are the same as in Table 2.

^*^p < 0.1; ^**^p < 0.05; ^***^p < 0.01.

##### 4.4.2.1 Gender

The individual-environment interaction theory posits that developmental outcomes are shaped by the interplay between individual traits and environmental factors (Lerner et al., 2006). Compared to Male, female exhibit greater dependence on parents and require more parental companionship and understanding (Duan et al., 2018). Therefore, gender differences may exist in the impact of early childhood grandparenting on deviant behaviors among rural junior high students. The regression results in Table 8 indicate that the effect of early childhood grandparenting on deviant behaviors does exhibit gender heterogeneity, with its influence primarily affecting females.

##### 4.4.2.2 Only-child status

Only-child and non-only-child families may differ in resource allocation and parental attention, which could potentially moderate the consequences of grandparenting. However, the regression results in Table 8 show that the coefficient for only-child is 0.445 and for non-only-child is 0.396. Both are positive and statistically significant, but the coefficient values are close. Further statistical tests (e.g., coefficient difference tests) indicate no significant difference between the two groups. This suggests that the effect of early childhood grandparenting on deviant behaviors does not differ between only-child and non-only-child rural junior high students. Regardless of only-child status, grandparenting significantly increases deviant behaviors, but the magnitude of this effect is similar across the two groups.

##### 4.4.2.3 Early family economic conditions

Family economic conditions play a significant role in the formation of individual human capital (Heckman, 2008). Households with favorable economic circumstances can provide greater financial support for children’s development and offer parents or grandparents greater material security, enabling them to engage more in the education of rural junior high school students. Conversely, impoverished families, constrained by limited resources, may neglect their children’s upbringing. Therefore, this study categorizes early economic conditions into favorable and unfavorable groups for separate regression analysis. The regression results indicate that early childhood grandparenting exerts a greater influence on rural junior high students from poorer families. This may stem from the fact that in economically disadvantaged households, grandparents, preoccupied with sustaining their own livelihoods, tend to focus primarily on meeting their grandchildren’s basic material needs rather than investing in their educational development (Sun and Yin, 2025). Consequently, these students exhibit higher rates of deviant behavior.

##### 4.4.2.4 Receiving preschool education

Preschool education serves as the foundation of national education, significantly impacting rural junior high students’ academic performance, non-cognitive skills, and behavioral habits (Liu et al., 2025). It generates cumulative positive effects throughout an individual’s lifetime (Tang et al., 2026). Early childhood grandparenting may result in some degree of educational deficiency within the family. Concurrent preschool education could potentially compensate for this educational gap caused by intergenerational caregiving, thereby mitigating its negative consequences. To examine this hypothesis, this study grouped participants based on preschool education exposure. Findings indicate that the adverse effects of early childhood grandparenting are more pronounced among rural junior high students who did not receive preschool education compared to those who did. This partially validates the claim that preschool education can compensate for deficiencies in family education and exert a corrective effect on deviant behaviors among rural junior high students.

## 5 Conclusion

Although a growing body of research has examined the immediate effects of grandparenting on child development, empirical evidence on its long-term associations with adolescent deviant behaviors remains limited. Using nationally representative data from the CEPS, this study investigated the impact of early childhood grandparenting on deviant behaviors among rural junior high students in China, with a particular focus on the mediating mechanisms derived from Problem Behavior Theory. First, early childhood grandparenting exerts a significant negative influence on deviant behaviors among rural junior high students. The robustness of these findings is confirmed through propensity score matching, a dual-robust model, and omitted-variable tests. Second, the impact of early childhood grandparenting on deviant behaviors exhibits significant heterogeneity across gender, early family economic conditions, and preschool education status. Its effects are more pronounced among female students, those from families with poorer early economic conditions, and rural junior high students who did not receive preschool education. Third, regarding the mechanisms, our results support both H2a and H2b. The perceived environment system (parent–child relationships and communication) and the personality system (future-oriented confidence and self-control) jointly mediated more than 80% of the total effect. Interestingly, future-oriented confidence emerged as the largest mediator, suggesting that early childhood grandparenting may undermine adolescents’ belief in their ability to achieve long-term goals, thereby increasing their susceptibility to deviant behaviors as a form of short-term gratification. This finding extends prior PBT research by identifying future-oriented confidence as a key pathway linking early family experiences to adolescent outcomes. Parent–child communication also played a substantial mediating role (23.01%), highlighting the importance of ongoing dialog between parents and children even when parents are not the primary caregivers.

In summary, this study makes three core contributions. First, it provides robust causal evidence for the long-term detrimental effects of early childhood grandparenting on adolescent deviant behaviors. Second, integrating and comparing the perceived environment and personality systems within PBT, it identifies future-oriented confidence as the strongest mediator, refining theoretical understanding. Third, heterogeneous effects (the protective role of preschool education, and higher vulnerability of girls and economically disadvantaged children) offer actionable insights for policy and practice.

## 6 Implications

This study highlights the significant and heterogeneous impact of early childhood grandparenting on deviant behaviors among rural junior high students, varying by gender, family economic status, and preschool education exposure. To address these challenges, families should prioritize strengthening parental involvement and enhancing parent–child communication, fostering emotional bonds that mitigate negative influences. Schools must actively engage in home-school collaboration, providing personalized guidance and promoting scientific educational concepts to compensate for family educational deficiencies. At the societal level, expanding access to high-quality early childhood care services and preschool education is essential. These measures can offset the adverse effects of intergenerational caregiving, promote healthy adolescent development, and contribute to the overall improvement of rural human capital, aligning with China’s rural revitalization strategy.

## 7 Limitations and future research

This study has several limitations. First, the use of cross-sectional data captures only a snapshot in time, which precludes the establishment of causal relationships. Second, the research focuses solely on junior high students, limiting the generalizability of the findings to other age groups. Third, the analysis of mechanisms may have overlooked other important mediating factors, such as parenting knowledge and parenting behaviors. Furthermore, this study mainly relies on students’ self-reported data for variable measurement, and some key variables (e.g., future-oriented confidence) are measured with a single item, which may introduce biases such as recall bias and social desirability bias, as well as concerns regarding reliability and validity. Future research should employ more refined scales for variable measurement, utilize longitudinal data to explore causal pathways, expand the sample to include a broader age range, and investigate other potential mechanisms contributing to deviant behaviors among rural adolescents.

## Data Availability

Publicly available datasets were analyzed in this study. This data can be accessed through http://ceps.ruc.edu.cn/.
