# Supplementary material for: The impact of early childhood grandparenting on deviant behaviors among rural junior high school students
Source: Front Psychol. 2026 Jun 22;17:1819600. doi: 10.3389/fpsyg.2026.1819600 (PMC13333468; doi:10.3389/fpsyg.2026.1819600)
Supplement: Supplementary file 1 [file Supplementary_file_1.docx]

**Appendix**

**TableA1 Balance Test (nearest neighbor matching)**

| Variable | Unmatched/  Matched | Mean | |  |  | t-test | |
| --- | --- | --- | --- | --- | --- | --- | --- |
|  |  | Treated | Control | Bias % | Reduct  bias % | *T-value* | *P-value* |
| male | U | 0.511 | 0.524 | -2.7 |  | 0.780 | 0.437 |
|  | M | 0.510 | 0.508 | 0.4 | 85 | 0.090 | 0.924 |
| Age | U | 13.313 | 13.379 | -13.9 |  | 4.000 | 0.000 |
|  | M | 13.313 | 13.301 | 2.5 | 81.8 | 0.620 | 0.537 |
| Only-Child | U | 0.294 | 0.265 | 6.5 |  | 1.900 | 0.057 |
|  | M | 0.294 | 0.299 | -1.1 | 82.5 | 0.270 | 0.791 |
| Self-Rated Health | U | 3.772 | 3.847 | -8 |  | 2.330 | 0.020 |
|  | M | 3.772 | 3.759 | 1.4 | 82.1 | 0.350 | 0.729 |
| Boarding | U | 0.482 | 0.465 | 3.5 |  | 1.020 | 0.307 |
|  | M | 0.482 | 0.499 | -3.5 | -0.2 | 0.830 | 0.405 |
| Receiving preschool education | U | 0.774 | 0.782 | -2 |  | 0.580 | 0.565 |
|  | M | 0.774 | 0.770 | 1 | 51.2 | 0.230 | 0.821 |
| Parental Educational Expectations | U | 16.498 | 16.345 | 4.6 |  | 1.340 | 0.180 |
|  | M | 16.499 | 16.335 | 4.9 | -6.6 | 1.170 | 0.241 |
| Father's level of education | U | 9.164 | 9.079 | 3.7 |  | 1.070 | 0.284 |
|  | M | 9.167 | 9.122 | 1.9 | 47.7 | 0.450 | 0.650 |
| Mother's level of education | U | 8.489 | 8.390 | 3.6 |  | 1.040 | 0.297 |
|  | M | 8.489 | 8.409 | 2.9 | 19.5 | 0.640 | 0.519 |
| Early Family Economic Conditions | U | 2.585 | 2.613 | -4.1 |  | 1.190 | 0.234 |
|  | M | 2.585 | 2.588 | -0.4 | 90.6 | 0.090 | 0.928 |
| Parental Relationship | U | 0.850 | 0.867 | -5 |  | 1.480 | 0.139 |
|  | M | 0.850 | 0.872 | -6.5 | -29.3 | 1.550 | 0.120 |
| two-parent families | U | 0.638 | 0.843 | -48.1 |  | 15.000 | 0.000 |
|  | M | 0.638 | 0.637 | 0.3 | 99.5 | 0.050 | 0.956 |

**TableA2 Joint Balance Test Results**

| Sample | Pseudo R² | LR chi2 | P>chi2 | Mean Deviation | Median  Deviation | B value | R value |
| --- | --- | --- | --- | --- | --- | --- | --- |
| Before matching | 0.129 | 653.67 | 0.000 | 3.9 | 2.7 | 90.0* | 1.31 |
| Nearest Neighbor Matching | 0.001 | 2.65 | 0.998 | 1.5 | 1.1 | 6.9 | 1.01 |
| Radius Matching | 0.000 | 0.37 | 1.000 | 0.7 | 0.5 | 2.6 | 0.96 |
| kernel matching | 0.001 | 4.08 | 0.982 | 1.9 | 2 | 8.5 | 1.19 |
| mahalanobis distance matching | 0.002 | 6.47 | 0.891 | 2.3 | 2.3 | 10.7 | 1.33 |

| 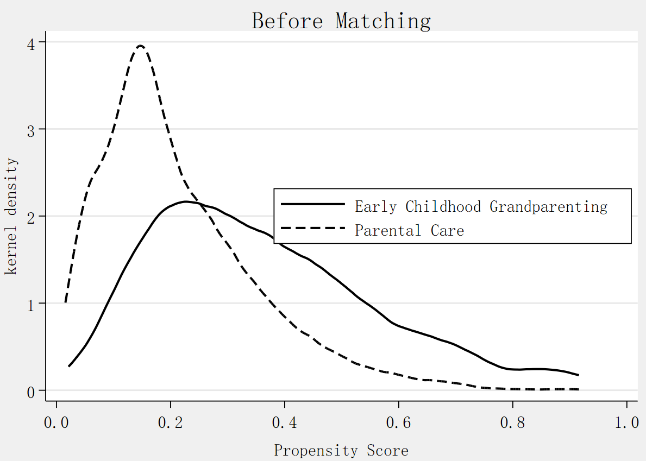  **Figure A1 Propensity distributions of treated and control group before matching** | 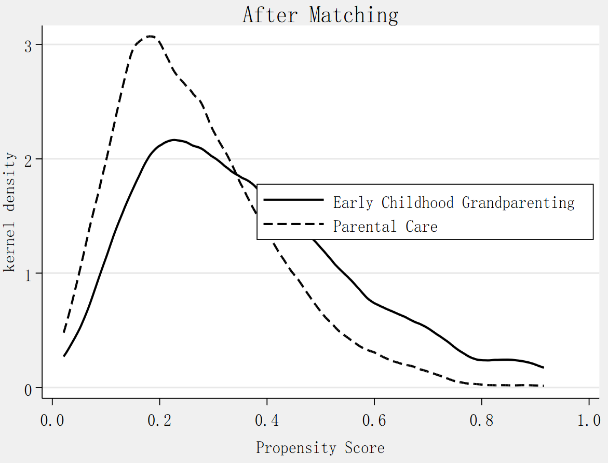  **Figure A2 Propensity distributions of treated and control group after nearest neighbor matching** |
| --- | --- |

| 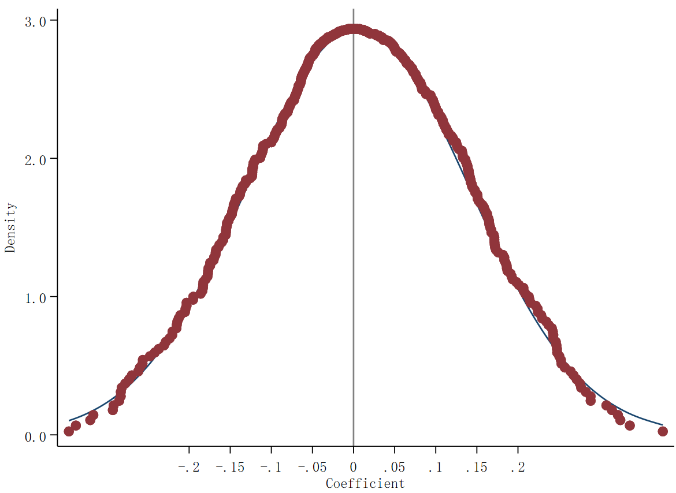  **Figure A3 Nonparametric Permutation Test** |
| --- |
